# Dermatomyositis Associated with Lung Cancer: A Brief Review of the Current Literature and Retrospective Single Institution Experience

**DOI:** 10.3390/life13010040

**Published:** 2022-12-23

**Authors:** Walid Shalata, Jeremy Zolnoorian, Amichay Meirovitz, Kim Sheva, Ashraf Abu Jama, Omar Abu Saleh, Alexander Yakobson

**Affiliations:** 1 Soroka Medical Center, The Legacy Heritage Center and Larry Norton Institute, Ben Gurion University, Beer Sheva 84105, Israel; 2 Medical School for International Health, Ben Gurion University of the Negev, Beer Sheva 84105, Israel; 3 Department of Dermatology and Venereology, Emek Medical Centre, Afula 18341, Israel

**Keywords:** dermatomyositis, lung cancer, paraneoplastic syndrome, dermal toxicity

## Abstract

Dermatomyositis is a rare inflammatory myopathy that is often related to lung cancer. In this retrospective observational study, we analyzed data from patients diagnosed with lung cancer at Soroka University Medical Center between January 2017 and July 2021. A total of 689 patients with lung cancer were included in this study, 97 of whom had small cell lung cancer and 592 had non-small cell lung cancer. We identified a single patient (60-year-old female) who presented with signs and symptoms of dermatomyositis, which was later confirmed to be associated with lung cancer as a paraneoplastic syndrome. Both our study and a recent review of the literature illustrate the temporal link between dermatomyositis and lung cancer, as well as reinforce the need for heightened cancer screenings in DM patients.

## 1. Introduction

Dermatomyositis (DM) is a rare autoimmune inflammatory myopathy characterized by proximal muscle weakness, mainly in the shoulders and hip girdle [1]. Various associated cutaneous pathologies have also been reported, including the heliotrope rash, which is a violaceous eruption on the eyelids, and Gottron’s papules, which are scaly erythematous papules on the dorsal aspect of the hands. DM is typically triggered by drugs or infectious agents, however, its occurrence as a manifestation of an underlying malignancy has been well documented. Studies show that 14.8% of patients with DM have an associated neoplasm [2]. The relative risk of carcinoma in these patients ranges between 3 and 8%, with risk increasing with age [3]. Several case studies have reported that DM is a potential paraneoplastic complication of lung adenocarcinoma particularly. Different malignancies have been reported including ovarian, breast, prostate, kidney, and hematological malignancies, but lung and gastrointestinal neoplasms have been the most common.

The relationship between dermatomyositis and cancer has a long history, first being suspected in 1916 when the disease was noted to co-occur with a case of stomach cancer. This suspected relation however was only confirmed in 1976 in a study assessing 258 cases of DM associated with various cancers [1]. Since then, the incidence of malignancies occurring in DM sufferers was reiterated in two large studies. The first was a study performed in 1992 in Sweden which revealed a 15% incidence of malignancy in DM patients [4]. The second, an Australian study, enrolled 537 patients in 2001, who had either polymyositis or DM, and revealed that malignancy occurrence either before, during, or after myositis diagnosis was 42% in DM sufferers. Overall, a six-fold risk for malignancy development in DM patients compared to the general population was revealed [5].

Lung cancer currently remains the leading cause of cancer-related deaths despite medical advances. In 2020, there were an estimated 1.76 million lung cancer-related deaths, accounting for 18.4% of total cancer deaths and making lung cancer the most frequent cause of cancer-related death globally [6]. The 5-year survival rate of lung cancer in the United States in 2019 was 19.4%, with the prognosis heavily influenced by the stage at which diagnosis is made [6,7]. The majority of cases are diagnosed once the cancer has metastasized beyond the lung. Diagnosis is made predominantly in individuals 70 years of age and older. The most predominant risk factor for lung cancer development is tobacco smoking, and in the United States where the number of smokers has declined, a positive influence on lung cancer incidence and mortality can be seen. However, the rates of lung cancer have continued to climb in developing countries throughout the world, largely due to the prevalence of smoking. The WHO estimates that 48% of men and 10% of women globally are smokers [8]. For this reason, disease prevention largely focuses on smoking avoidance and cessation.

Mutations in the epidermal growth factor receptor (*EGFR*) gene have been implicated in the incidence of lung adenocarcinoma. Approximately 10-30% of patients demonstrate a specific genetic profile regarding these mutations. Physiologically, EGFR promotes cell proliferation, differentiation, and apoptosis of normal cells [8]. Mutations in *EGFR* lead to increased downstream signaling and uncontrolled cellular proliferation and growth. The protein encoded by the *EGFR* gene acts as a transmembrane glycoprotein, which is part of the v-erb-b2 erythroblastic leukemia viral oncogene (Erb/B) human epidermal receptor (HER) family of receptor tyrosine kinases [9]. Tyrosine kinase inhibitors are therefore able to block EGFR-derived signal transduction and halt cell growth and differentiation in patients with *EGFR* mutations [10]. *EGFR* mutations play a crucial role in the pathogenesis of lung adenocarcinoma and affect the ability of targeted therapies to improve patient prognoses.

Even though the connection between the two diseases has been documented, it remains exceedingly rare. Soroka University Medical Center is the primary source of medical care for the southern half of Israel, yet few if any cases are documented. We performed a search of all the digitized hospital oncology records for patients diagnosed with lung cancer and who also had an associated DM diagnosis. Here, we present the only patient at our medical center in the last 4 years whom we were able to identify as having DM associated with lung cancer. In addition, we compiled a list of all similar case studies we could find in the literature from the last 10 years, a total of 18 patients. Included are all available characteristics we could gather about their respective cases, as well as a literature review on the current pathophysiology, signs, symptoms, and classification of DM and associated lung cancer.

While the association between DM and lung cancer is by no means novel, our motivation was to add to the significantly limited data and statistics in the literature on the link between the two diseases. Our case study illustrates the full clinical course from disease discovery to the creation and implementation of a treatment plan. We hope our findings and data collection may help future teams establish a more direct link or discover new revelations in this area. The following case report and associated literature review provide evidence of the importance of the use of cutaneous signs in the discovery of underlying malignancy.

## 2. Materials and Methods

This is a single-institution retrospective, observational study without intervention. The study was approved by the Institutional Review Board of Soroka Medical Center (approval no.0316; on 2 December 2021).

The study included patients admitted to Soroka Medical Center between January 2017 and July 2021.

- The inclusion criteria for the study were:
- Patients aged 18 years or older.
- Patients diagnosed with lung cancer (local (stage 1–3) or advanced or metastatic disease (stage 4)).

Patients must have been treated only in Soroka Medical Center or have a full follow-up history in Soroka Medical Center’s records.

Each of the study patients was presented and discussed with a multidisciplinary medical team when admitted to Soroka Medical Center’s Oncology Institute as per standard protocol. This team includes a general medical and radiation oncologist, an imaging and nuclear physician, a pulmonologist, a pathologist, and a thoracic surgeon. The team discussion is based on patient status, pathology, and imaging. Each patient is assigned a primary physician who is responsible for the treatment course.

Patients with previous advanced or metastatic diagnoses are treated mainly by medical oncologists, and the treatment plan is generally based on National Comprehensive Cancer Network (NCCN) recommendations. Routine molecular profiling is performed for each patient when possible.

**Figure 1:**
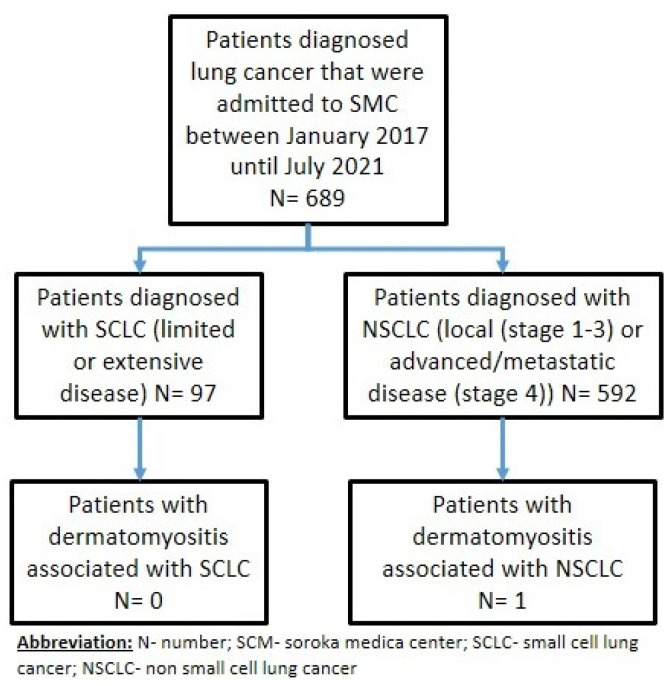
The workflow used for this single-center, retrospective, observational study that revealed dermatomyositis associated with lung cancer in a single patient.

We identified a single patient (through interviews with oncologists and dermatologists and patient databases) with dermatomyositis that was associated with lung cancer (Figure 1).

Multiple literature searches were performed using PubMed to obtain the studies referenced in this article. Searches were performed from 20 August 2022 to 30 September 2022. Search terms included “Lung Cancer (AND) Dermatomyositis” in the Title/Abstract. The search terms were chosen to best find the broadest range of published papers on the topic of dermatomyositis associated with lung cancer. The initial search returned 54 results. From the search results, we reviewed all the papers displayed and found 17 relevant case studies and literature reviews, with 19 patients in total for comparison, including one patient from our own clinic.

All case reports were synthesized into a table. Of the case reports, all were published between the years 2012 and 2021, with the majority published between 2014–2019. Case studies were segregated based on the specific type of lung cancer, stage of lung cancer, presence of activating mutations, and treatment type received. Trends were enumerated qualitatively through a table (Appendix A).

## 3. Exclusive Case Study

A 60-year-old female was referred to the emergency room of Soroka Medical Center in January 2020 by a primary care physician due to a rash on her hands and face, accompanied by itching and weakness. The patient had no history of chronic disease or chronic medication. She never smoked. She had no family history of cancer.

The patient reported that she had suffered from redness accompanied by itching and pain on the face and fingers for the past two years. An accompanying rash had then appeared which spread to the forearms, abdomen, arms, and knees. The patient experienced pain in the arms and legs with muscle weakness, had difficulty walking, and was unable to raise her hands. In addition to a weight loss of 14 kg over the past year, the patient suffered from night sweats (without an accompanying fever), and difficulty and pain when swallowing.

**Figure 2:**
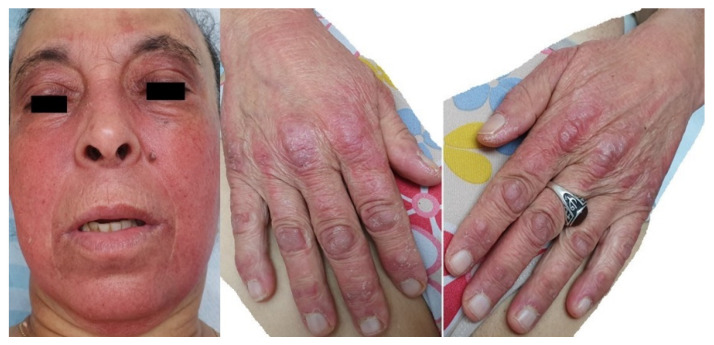
Dermatomyositis symptoms presenting on the face, palms, and around the nail base.

A physical examination revealed a rash in the upper thoracic region consisting of arithmetic carpets and a V-neck sign. On the upper back and occiput, there was a rash consisting of arithmetic, flat carpets, and a shawl sign. Poikiloderma was noted on the occiput, upper thorax, bilateral arms, and bilateral waist, with hyper-pigmentation spots on slightly arithmetic skin. Additional presenting symptoms were noted on: Face- on the forehead, eyelids, cheeks, and around the eyes, a rash consisting of oval pink carpets with a sharp border on a slightly edematous skin background was present; Scalp—impression of thinning hair; Upper limbs—elbows lateral part bilateral arithmetic papules with an irregular border covered with little scales; On the palms of the hands (dorsal area) purple-pink papules (Gottron papules) accompanied by minor scaling in metacarpophalangeal (MCP), distant interphalangeal (DIP), proximal interphalangeal (PIP) regions; Palms—using dermoscopy—dilation of blood vessels around the nail base; Toenails—onychodystrophy—a loss of shine and yellowing (Figure 2). No enlargement of the lymph nodes was noticed. Lung and cardiovascular evaluations were normal, and the electrocardiogram was unremarkable. Routine laboratory investigations (complete blood count and biochemical profile) showed no abnormalities.

The patient was hospitalized for further investigations. Laboratory investigations, including complete blood count, biochemical profile, rheumatologic tests, oncologic markers, tuberculosis test, urine tests, and stool tests, showed no abnormalities (Table 1) The spirometer flow volume test showed normal lung function. Gastroscopy and colonoscopy tests revealed no pathological findings. A multidisciplinary conference, including a dermatologist, rheumatologist, and neurologist, concluded that the patient presented with a clinical picture matching that of dermatomyositis, but that further evaluations should be considered. A skin biopsy was performed where histopathological results supported a diagnosis of dermatomyositis (Table 2).

The patient received promethazine (25 mg) daily to relieve itching, and topical creams for the dermatomyositis, however, minimal benefits were rendered.

Total body computed tomography (CT) showed a mass in the left upper lung (LUL) (1.7 cm in diameter) resulting in a differential diagnosis of a fibrotic process, space-occupying lesion (SOL), multiple small lung nodules suspected to be metastasis, and lymphadenopathy of the left supraclavicular area and mediastinum (1.4 cm in diameter).

A supraclavicular lymph node excision biopsy was done, and pathological results revealed adenocarcinoma of lung origin.

**Figure 3:**
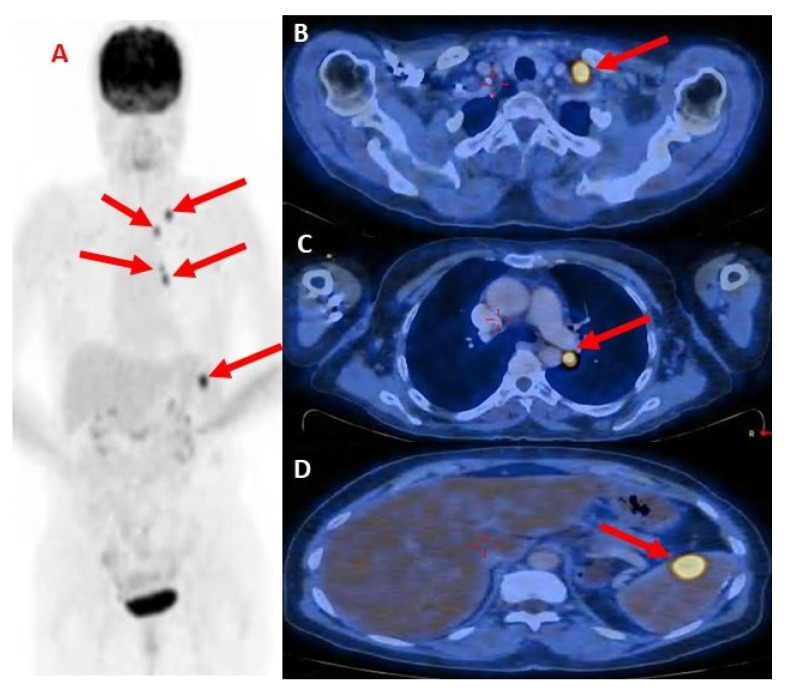
(**A**) PET-CT scan showing the spread of metastatic disease; (**B**) showing hyper-metabolic uptake in the LUL mass; (**C**) Hypermetabolic uptake and enlargement of the lymph nodes in the left supraclavicular area; and (**D**) hyper-metabolic uptake in the spleen. Red arrows indicate metastasis.

Positron emission tomography-computed tomography (PET-CT) showed hyper-metabolic uptake in the LUL mass (1.7 cm in diameter) and multiple metastases in the mediastinal lymph nodes and the hilum of the left lung. Hyper-metabolic uptake and enlargement of the lymph nodes in the left supraclavicular area (1.1 cm in diameter) and a hyper-metabolic uptake in the spleen (1.2 cm in diameter) were also seen (Figure 3). Magnetic resonance imaging (MRI) of the head showed no evidence of metastatic disease. The presumptive clinical diagnosis was stage T1 N3 M1 (stage 4) non-small cell lung cancer. Molecular testing showed programmed death ligand (PDL)-1 staining at 75%, positive for EGFR mutations (p.Glu746_Ser752delinsVal.), and was negative for ALK rearrangement and BRAF.

**Figure 4:**
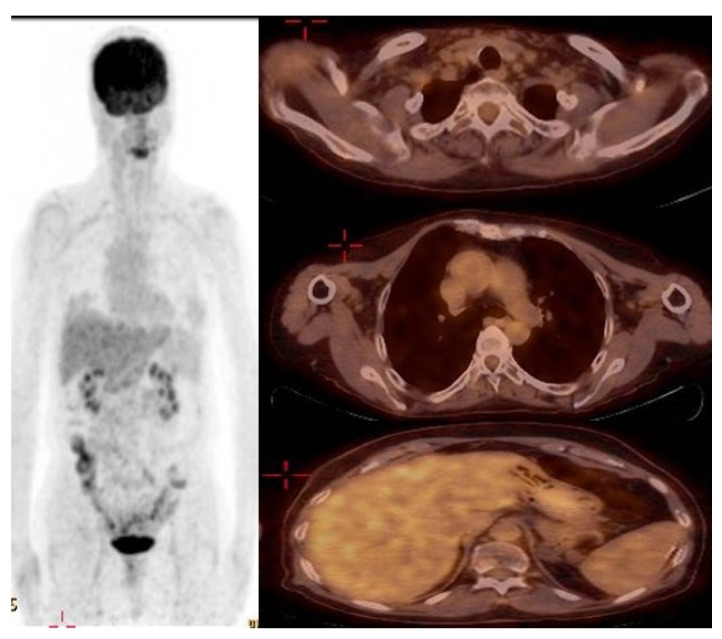
PET-CT showing complete radiologic response to Osimertinib treatment.

Following one month of Osimertinib administration (80 mg once a day), the patient’s dermatological symptoms improved, and two months later a PET-CT showed a radiological significant response (Figure 4). The left supraclavicular lymph nodes had receded in diameter with no pathological uptake. The mediastinal lymph nodes and the hilum of the left lung returned to normal size and were without pathological uptake. The LUL lesion had receded with lower pathological uptake. The splenic lesion had decreased in size without pathological uptake. (High muscle absorption was demonstrated due to a lack of fasting as required).

A multidisciplinary conference including an oncologist, dermatologist, rheumatologist, and neurologist concluded that the patient presented with a clinical picture of dermatomyositis as a paraneoplastic syndrome, and therefore needed to continue the Osimertinib treatment (80 mg once a day daily). On the last follow-up (10/2022) the patient was still on treatment without recurrence of the dermatomyositis.

## 4. Dermatomyositis

### 4.1. General Information

Dermatomyositis (DM) is a subset of a larger group of diseases known as idiopathic inflammatory myopathies (IIM) that affect the muscles, skin, lungs, and joints. DM is characterized by specific skin findings, often accompanied by variable systemic symptoms [10]. DM is the most common of the IIMs, with an estimated prevalence of 1–6 per 100,000 adults in the United States [11]. DM affects women more often than men in a 2:1 ratio [12].

### 4.2. Pathophysiology

DM is a complex, multifactorial disease involving both immune and non-immune mechanisms. As an inflammatory disorder, T cells and auto-antibodies present as key players in DM manifestations, with genetic factors also playing a role. Non-immune mechanisms appear to involve pathogenic environmental factors. While research regarding the pathophysiology of this intricate disease is continuously ongoing and revealing further insight, unfortunately at this time, the exact pathophysiology of DM is yet to be completely understood [12,13].

### 4.3. Dermatological Signs

**Figure 5:**
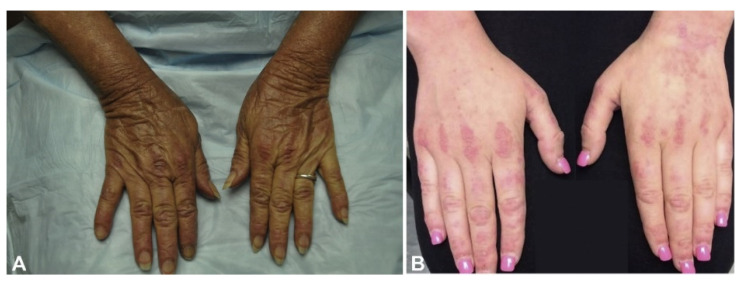
Gottron’s papules (**A**) and the Gottron’s sign (**B**) on the dorsal surfaces of the hands of two patients with dermatomyositis [12].

**Figure 6:**
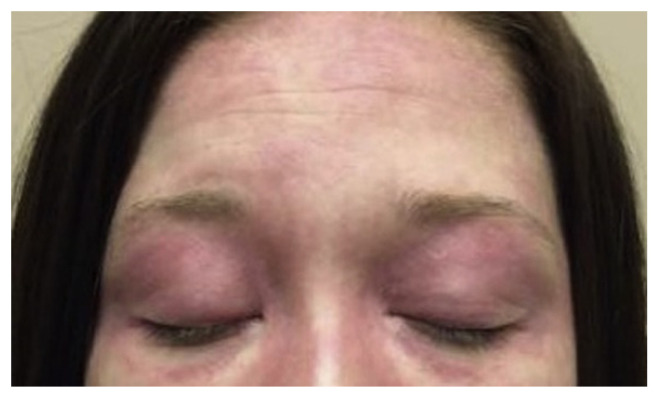
Heliotrope rash on the face of a patient with dermatomyositis [12].

**Figure 7:**
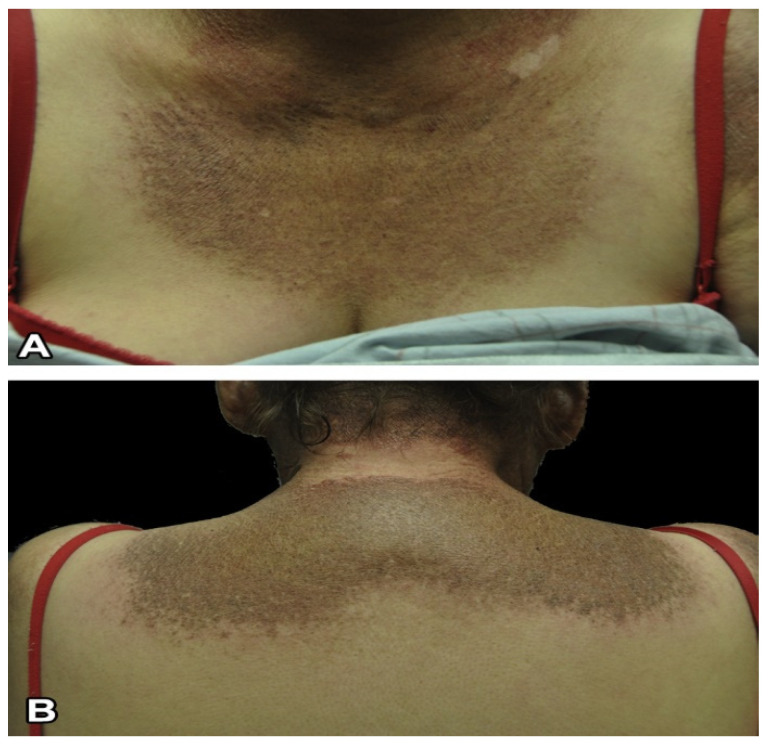
V sign (**A**) with erythema and poikiloderma and shawl sign (**B**) in a patient with dermatomyositis [12].

**Figure 8:**
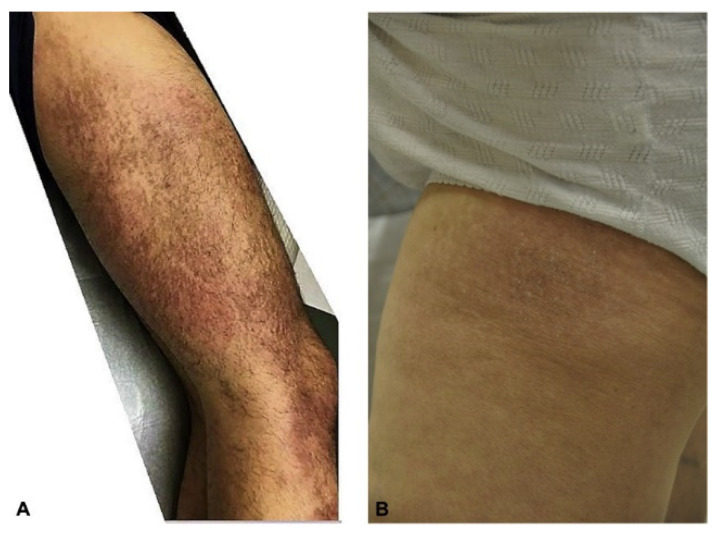
Holster sign on the (**A**) right lateral thigh and (**B**) right lateral hip of two patients with dermatomyositis [12].

The pathognomonic signs of DM are Gottron’s papules (Figure 5A), Gottron’s sign (Figure 5B), and the heliotrope rash (Figure 6) [12,13]. Slightly less common, but still highly associated with DM are changes in the nail folds, shawl sign, V sign (Figure 7A,B), Holster sign (Figure 8A), lower extremities (Figure 8B), and involvement of the scalp. Patients may present with multiple cutaneous manifestations, and the progression of skin lesions is often independent of myositis disease progression [11,13]. In the absence of distinct serology and biopsy evidence, at least one of the pathognomonic signs must be present for a DM diagnosis [13].

### 4.4. Muscle Manifestations

Around 80% of DM patients exhibit some level of myopathy, classically presenting as proximal muscle weakness [13]. Functional presentations may vary and can be broad ranging from trouble lifting items, walking or climbing stairs to dysphonia, dysphagia, and muscle pain [12]. Upon biopsy, muscle specimens are characterized by perifascicular atrophy, perimysial inflammation, and high levels of major histocompatibility complex (MHC) I [13]. Immunohistochemical staining for the MxA protein may potentially be a more sensitive test for DM than traditional pathological muscle biopsy analysis. Laboratory analysis may reveal a significant elevation of muscle enzymes (e.g.: serum creatine kinase). DM may also present with certain specific autoantibodies including Mi-2, MDA5, anti-TIF-1, NXP2, and SAE [12,13].

### 4.5. Classification of DM (Subtypes) (According to ENMC 2018 Dermatomyositis Classification Criteria) ^[13]^

A DM classification can be made if the following clinical and skin biopsy features are present *:

○ Clinical exam findings (at least two of the following): Gottron’s sign, Gottron’s papules, and/or heliotrope rash.
○ Skin biopsy findings: interface dermatitis.

* Based on established skin criteria that allow for classification of clinically amyopathic DM [13,14].

A DM classification can be made if the following clinical features are present with either DM muscle features ** or a DM-specific autoantibody ***:

○ Clinical exam findings (at least one of the following): Gottron’s sign, Gottron’s papules, and/or heliotrope rash.

** DM muscle features:

- Proximal muscle weakness.
- Elevated muscle enzymes

Suggestive DM muscle biopsy findings include lymphocytic infiltrates (often perivascular), evidence of perifascicular disease (perifascicular predominant fibers that are pale on cytochrome oxidase (COX) staining and/or positive on neural cell adhesion molecule (NCAM) staining).

Definitive DM muscle biopsy findings include perifascicular atrophy and/or perifascicular MxA overexpression with rare or absent perifascicular necrosis.

*** DM-specific autoantibodies:

- Anti-TIF1 γ
- Anti-NXP2
- Anti-Mi2
- Anti-MDA5
- Anti-SAE

Additional notes:

○ A classification of DM cannot be made in the absence of cutaneous DM features.
○ Patients with an anti-synthetase autoantibody will be classified as having anti-synthetase syndrome and not DM.
○ Anti-synthetase syndrome patients with a DM-like rash will be classified as having “anti-synthetase syndrome with a DM-like rash”.
○ Patients with anti-HMGCR or anti-SRP autoantibodies will be classified as having immune-mediated necrotizing myopathy and not DM.
○ Anti-HMGCR + patients with a DM-like rash will be classified as having “anti-HMGCR myopathy with a DM-like rash”.
○ Anti-SRP + patients with a DM-like rash will be classified as having “anti-SRP myopathy with a DM-like rash”.
○ Patients with a DM-specific autoantibody will be subclassified according to that autoantibody (e.g., anti-TIF1 γ DM, anti-NXP2 DM).
○ Patients who have DM without a DM-specific autoantibody will be subclassified as having “autoantibody negative DM”.
○ Ulcerating lesions on the extensor surfaces of the metacarpophalangeal, proximal interphalangeal, and/or distal interphalangeal joints (as may be seen in anti-MDA5 DM) are considered equivalent to Gottron’s papules.

## 5. Dermatologic Paraneoplastic Syndromes Associated with Lung Cancer

### 5.1. General Information

Paraneoplastic dermatoses are often associated with internal malignancy, either as a result of direct cutaneous metastasis, or ectopic humoral syndromes. Lung cancer has several well-documented skin signs that may assist diagnosis and treatment (Table 3), as well as herald a general prognosis [15,16].

### 5.2. Dermatomyositis Associated with Lung Cancer

There is a well-established link between DM and malignancy, with occurrence rates between 3–40% of patients developing cancer [17,18]. Malignancy is the most common cause of death in DM patients, with 5-year survival rates of adult patients being between 60% to 90% [18]. The onset of both DM and associated cancer are often closely linked. While the exact mechanism of this correlation is still being investigated, several plausible theories have emerged. Immunosuppressive treatment for DM may allow for the incidence of malignancy; immunological response to malignancy may induce DM, or increased workup and imaging of DM patients may increase the detection rates of cancers [18]. DM may be considered a paraneoplastic disorder in this regard, with signs and symptoms often regressing with the treatment of cancer and reemerging with relapse of the disease [9].

## 6. Discussion

DM is a rare disorder that occurs more frequently in females than males (2:1). DM also displays a bimodal distribution, with incidence peaking between ages 5–15 and 50–60 [19]. Interestingly the relatively recent case reports reviewed in this manuscript did not reflect either of these factors, with patients being almost evenly divided based on sex, with 55% male and 45% female, and the youngest patient being 48 years old (Appendix A). A tabulated review by Fujita et al. of patients with lung cancer associated with DM occurring between the years of 1947–2000 also did not concur with these statistics, with 76.2% being male and the youngest patients being 44 years old (one male, one female) [20]. This may be due to a more specific subset of the DM population being examined (patients diagnosed with both DM and lung cancer), which may consist of a differential sex distribution, or due to the limited patient cohort which may not be accurately reflective of the larger population. In addition, while juvenile DM has been described frequently in the literature, it seldom presents with lung carcinoma [21].

The link between DM and cancer has been well established in the literature, and while the specific types vary between populations, lung cancer is frequently observed [5,22,23,24,25,26,27,28,29,30,31,32]. The majority of cancer cases occur within 1 year prior to or post DM diagnosis, and this was observed in our data set, with all but 1 patient having both diagnoses in this timeframe.

Most DM diagnoses were made at the same time as the lung cancer diagnoses (65% of the cohort). This raises several questions regarding the link between the two diseases, including how long one or both diseases occurred before the patient presentation or clinical diagnosis, and whether earlier or more frequent screening for either disease could lead to earlier diagnoses or improved outcomes. Several studies recommend the use of more aggressive cancer screening in newly diagnosed DM patients [10,17], again reiterating the link between DM and cancer. Research is ongoing concerning the exact screening guidelines for optimal outcomes and whether there are more specific DM screening modalities that should be used.

There is new evidence suggesting that certain DM-associated autoantibodies may play a role in cancer risk [5,9,18]. Antibodies targeting the nuclear matrix protein (NXP-2) and transcription intermediary factor 1γ (TIF-1γ) are highly specific to DM and one study has suggested a possible association between anti-NXP-2 and anti-TIF-1γ autoantibodies and cancer, neither of which was statistically significant [25]. Further research is needed to elucidate this potential connection, which may be a future avenue for both DM and DM-associated cancer screening, as well as provide insight into the nature of the causal pathway between the two diseases.

The exact link between DM and lung cancer is an active area of research. Current theories include the fact that immunosuppressive treatment for DM may allow for cancer emergence; cancer may bring about immunological changes that lead to DM; certain DM-associated autoantibodies may play a role in cancer promotion/progression; once a diagnosis of DM or lung cancer is made, the patient is under increased surveillance resulting in the diagnosis of the second disease [27]. Malignancy is a leading cause of death in DM patients, with the 5-year survival rate of DM patients being between 60–90.1%, and the 5-year survival rate for DM patients with cancer being between 10–56% [30,31,32]. Interestingly, to the best of our knowledge, there is only a single published case of DM that resulted in disease resolution following treatment with immunotherapy [32]. The large majority of cases report DM as an adverse effect of ICI (pembrolizumab -carcinoma of unknown origin, squamous cell carcinoma of lung and lung adenocarcinoma; atezolizumab-in small cell lung cancer and non-small-cell lung cancer; nivolumab- lung adenocarcinoma and ipilimumab- small-cell lung cancer) [33,34,35,36,37,38,39]. Bendewald et al. have reported that the estimated incidence of DM is 9.63 cases per million people [22]. This highlights just how rare this disease is without even considering its co-presentation with lung cancer. It is thus of significant importance to assess, study and report all cases to elucidate insight into the disease’s exact mechanisms and links to lung cancer. The recommendations for cancer screening remain pertinent for DM patients especially if they are suffering from the skin manifestations of TIF-1γ DM, which presents with a darker red rash, that is scattered all over the body. In addition, if skin manifestations are severe and/or complicated by dysphagia (in the absence of an interstitial lung abnormality (ILA)), and are not responding to corticosteroids, TIF-1γ antibody levels should be checked and cancer screening should be performed to rule out any potential malignancy involvement [22,23]. Lastly, in such cases, a multidisciplinary conference should be held for optimal patient treatment outcomes.

Limitations of this study include firstly, that many patients were not followed for a long period, where of the patients that died, all were within 1 year of both diagnoses (25% of cohort, range of 1–11 months). Secondly, unfortunately, patient databases only became fully digitized at Soroka Medical Center in 2017 and therefore the retrospective analysis for this study was restricted to patient data only collected from 2017 onwards. Lastly, the patient databases used in this study were those under the jurisdiction of the Oncology Department. There may be patient data that was missed due to the fact that these patients were logged in alternative department databases as patients initially suffering from DM that then developed lung cancer, instead of being primary oncology patients. Other limitations are that our study does not examine or compare different treatment protocols and outcomes. More recent studies looking at DM and DM-associated lung cancer have taken a more personalized approach to the categorization of DM, and with that more personalized treatment plans. While this is a fascinating and promising field of future research, we felt it was outside the scope of our current paper.

Future studies could examine the exact mechanism of disease causation and progression, as well as the differences in unique DM subtypes. Increasingly more studies are looking at the different genetic makeups of DM and what specific mutations and autoantibodies are present. It would be very interesting to see how the whole clinical picture differs in these patients, from disease onset and skin manifestations to overall treatment and prognosis. It’s plausible to think that treatment may even differ and have different outcomes depending on the mutations at play. Additionally, a more comprehensive future study could examine patient populations from a bidirectional approach, following lung cancer patients that develop DM as well as DM patients that develop lung cancer.

## 7. Conclusions

Dermatomyositis is a rare autoimmune myositis that is frequently associated with malignancy. Our study and literature review reinforce both the association between dermatomyositis and lung cancer and the need for heightened surveillance of dermatomyositis patients. In particular, clinicians should have an elevated level of suspicion with newly diagnosed dermatomyositis and should closely monitor cancer screening and the emergence or change of any skin signs as predictors of malignancy.

## Figures and Tables

**Table 1:** Patient laboratory test results.

Lab Test	Result	Lab Test	Result
ANA MULTIPLEX	Positive	ANTI CENTROM B. (<0.9 AI)	<0.2
ANTI DS DNA (5.00 IU/ML)	Negative	ANTI CCP	Negative
ANTIRIBOSOMAL P (<0.9 AI)	<0.2	RF (>30 IU/ML)	<20
ANTI RNP (<0.9 AI)	<0.2	P-ANCA	Negative
ANTI RNP-A (<0.9 AI)	<0.2	C-ANCA	Negative
ANTI SM (<0.9 AI)	<0.2	Myositis screen Ab 3	TIF-1 gamma possitive
ANTI SM/RNP (<0.9 AI)	<0.2	Myositis screen Ab 16	RO-52 positive
ANTI SCL-70 (<0.9 AI)	<0.2	Myositis screen Ab 11	SRP negative
ANTI RNP 68 (<0.9 AI)	<0.2	Myositis screen Ab 8	PM-Scl 100 positive
ANTI RO (SS-A) (<0.9 AI)	<0.2	Myositis screen Ab 3	TIF-1 gamma positive
ANTI LA (SS-B) (<0.9 AI)	<0.2	Myositis screen Ab 16	RO-52 positive
ANTI SS-A 52 (<0.9 AI)	<0.2	RF (>30 IU/ML)	<20
ANTI SS-A 60 (<0.9 AI)	<0.2	P-ANCA	Negative
ANTI CHROMATIN (<0.9 AI)	<0.2	C-ANCA	Negative
ANTI JO-1 (<0.9 AI)	<0.2		

**Table 2:** Skin biopsy results.

Indirect Immunofluorescence	Fibrinogen	C3	IgM	IgA	IgG
Negative	+3 linear, continuous deposits along the basement membrane.	Focal +2, continuous deposits along the basement membrane.	Linear +2, continuous deposits along the basement membrane.	Negative	Negative

**Table 3:** Paraneoplastic dermatoses of lung cancers.

Ectopic ACTH Syndrome	Dramatic Hyperpigmentation of the Skin, Due to Excessive ACTH Secretion by Tumor
Bronchial carcinoid syndrome	Episodic flushing of the face, chest, and neck for 1–30 min episodes. Believed to be caused by tumor release of serotonin and 5-hydroxytryptophan.
Erythema gyratum repens	Quickly spreading, pruritic rash on the trunk and spreading to extremities. Serpiginous, polycyclic morphology, often compared to “wood grain.”
Acanthosis nigricans maligna	Hyperkeratotic and hyperpigmented skin plaques, often found in the axilla, groin, and neck.
Acanthosis Palmaris	Velvety hyperkeratotic change of the skin of the palms. Said to resemble the stomach lining of bovines, hence its other name “tripe palms.”
Hypertrichosis lanuginosa	Unpigmented, long, thin hairs. When associated with malignancy, often progress cephalocaudally, starting on the face/forehead and progressing downwards.

## Data Availability

Data is contained within the article or are available from the authors upon reasonable request.

## Appendix A

**Table A1:** A summary of patients suffering from paraneoplastic dermatomyositis associated with lung cancer as reported in the literature from 2012–2022.

Sex	Age (Years)	Primary Tumor	Stage	Tumor Mutation (EGFR, ALK, ROS, BRAF)	History of Smoking	Skin Rash (Present/Absent)	DM Symptoms Prior to Diagnosis	Time to DM Resolution from Treatment	Ref.
Male	74	Lung adenocarcinoma	II-A	EGFR	Never	Present (Gottron’s sign)	Simultaneous	No resolution	[25]
Male	60	Small cell lung carcinoma	Unknown	None	80 pack/years	Present (heliotrope + Gottron’s sign)	Simultaneous	No resolution	[38]
Male	73	Lung squamous cell carcinoma	II-B	Not Verified	1 pack/day for the past 30 years	Present (heliotrope)	Before (2 months)	1 month	[39]
Male	57	Non-small cell lung carcinoma (type not reported)	Unknown	Not Verified	Not reported	Present	Simultaneous	After removal of tumor	[26]
Male	60	Lung squamous cell carcinoma	T2N2Mx	Not reported	40 pack/years	Present (heliotrope)	Simultaneous	No resolution	[40]
Female	61	Small cell lung carcinoma	IA	Not Verified	Yes (period not reported)	Absent (Gottron’s sign)	Before (6 months)	10 months	[41]
Female	63	Lung adenosquamous	IV	Not Verified	Not reported	Present (heliotrope)	Simultaneous	6 months	[42]
Male	69	Lung cancer (type not reported)	IB	Not Verified	1 pack/day for 40 years	Present (heliotrope + Gottron’s sign)	Simultaneous	After tumor removal	[43]
Male	54	Small cell lung carcinoma	Unknown	Not reported	40-year history	Present (heliotrope)	Simultaneous	4 weeks	[44]
Female	63	Small cell lung carcinoma	Unknown	Not reported	Not reported	Present (heliotrope)	After	No resolution	[45]
Female	51	Lung squamous cell carcinoma	IV	C-Met	Never	Present	Simultaneous	Not reported	[46]
Male	59	Lung adenocarcinoma	IV	Not reported	Not reported	Present (Gottron’s papules)	Simultaneous	No resolution	[47]
Male	63	Lung adenocarcinoma	unknown	Not reported	Not reported	Present (Gottron’s papules + heliotrope)	Before (8 months)	Unknown	[48]
Male	58	Lung adenocarcinoma	IV	Not reported	37 pack/years	Present (heliotrope rash + Gottron’s sign)	Simultaneous	Not reported	[20]
Female	48	Small cell lung carcinoma	IV	Not Verified	Never	Present (Gottron’s sign + papules)	Simultaneous	Not reported	[49]
Female	68	Small cell lung carcinoma	IIIA	Not reported	Not reported	Present (Gottron’s sign)	Simultaneous	Not reported	[50]
Female	82	Lung adenocarcinoma	IV	EGFR Exon-20 Insertion	Not reported	Present (heliotrope)	After (8 months)	Not reported	[51]
Male	68	Small cell lung carcinoma	unknown	RB1, P53, MYC	Yes (period not reported)	Present (shawl + v signs)	Simultaneous	4 months	[52]
Female	60	Lung adenocarcinoma	IV	EGFR	Never	Present (V-sign, Gottron’s sign)	Before (2 years)	1 month	Current work
